# P02-07 Physical activity promotion project: intervention and evaluation among pregnant women

**DOI:** 10.1093/eurpub/ckac095.026

**Published:** 2022-08-29

**Authors:** Shelly Ruart, Stéphane Sinnapah, Olivier Hue, Eustase Janky, Sophie Antoine-Jonville

**Affiliations:** 1 Sport Sciences Department Laboratory ACTES EA3596, Univ Antilles, Pointe-à-Pitre CEDEX, Guadeloupe; 2 Gynaecology, Obstetrics Department, University Hospital of Guadeloupe, Pointe-à-Pitre CEDEX, Guadeloupe

**Keywords:** barriers to physical activity, counseling, physical activity behavior, health outcomes, public health

## Abstract

**Background:**

We have set up a project to promote physical activity. Physical activity counseling was dispensed to aimed decrease perceived barriers and improve physical activity behaviors. The objective was to evaluate the process and the effectiveness for public health.

**Methods:**

A quasi-experimental trial was conducted in the Maternity Unit of a hospital in Guadeloupe between 2017 and 2018. 96 pregnant women were allocated to a control or intervention group. Physical activity counseling throughout pregnancy was dispensed to the women in the intervention group by trained health professionals. The physical activity levels (an activity monitor and PPAQ) and perceived barriers (questionnaire) were assessed in each trimester. The evaluation of the process consists of evaluating the frequency of counseling in accordance with the recommendations, reported by the women in the intervention group and their effect on physical activity behaviors compared control group. The counseling received was measured with a questionnaire.

Statistical Analysis: For intervention, repeated measures ANOVAs was used to investigate changes between groups over time for PA behaviors, perceived barriers and the neonatal outcomes collected monthly from delivery to 2 months. For evaluation of the process, Chi2 tests explored PA counseling received by group. Repeated measures ANOVAs were used to investigate the changes between groups over time for PA behaviors.

**Results:**

Firstly, the perceived barriers, such as a lack of information about the health benefits and risks and insecurity related to practice (all p < 0.05), were different in favor of the intervention group. There were no significant between-group differences for the major indices of PA, whether measured or reported, and perinatal health outcomes. Secondly, the evaluation of the process showed that women in the intervention group reported more frequently receiving PA counseling throughout their pregnancy vs. the control group (p < 0.001). The same results were found for counseling received in accordance with the recommendations, and women pre-pregnancy overweight (all p < 0.001). All women in the intervention group (normal and pre-pregnancy overweight) who reported received counseling throughout pregnancy reported larger total PA compared to women in the control group (all p < 0.05). However, the total quantity of PA was not different in women who received counseling in accordance with recommendations between the groups (all p > 0.05).

**Conclusions:**

The intervention was unable to limit the decline in PA or improve health outcomes. However, it was associated with an improvement in the perceived barriers. The evaluation of this project has highlighted encouraging signs for PA promotion.

